# Two‐Dimensional Accuracy of Provisional Laminate Veneers Designed Using Two Digital Smile Design Platforms and Fabricated by Additive and Subtractive Manufacturing

**DOI:** 10.1111/jerd.70178

**Published:** 2026-05-03

**Authors:** Murat Feriz, Zeliha Şanıvar Abbasgholizadeh, Yılmaz Umut Aslan, Yasemin Özkan

**Affiliations:** ^1^ Department of Prosthodontics, Faculty of Dentistry Marmara University Istanbul Türkiye; ^2^ Institute of Health Sciences Marmara University Istanbul Türkiye

**Keywords:** additive manufacturing, digital prosthodontic workflow, digital smile design, provisional laminate veneers, subtractive manufacturing, two‐dimensional accuracy

## Abstract

**Objectives:**

To evaluate and compare the two‐dimensional (2D) accuracy of provisional laminate veneer restorations designed using two digital smile design platforms and fabricated through additive and subtractive manufacturing techniques within a standardized digital workflow.

**Materials and Methods:**

This study included 25 participants for whom provisional laminate veneer restorations were digitally designed for the maxillary anterior region using two digital smile design platforms. Each digital design was subsequently fabricated using two manufacturing methods, additive manufacturing (stereolithography‐based printing) and subtractive manufacturing (milling), resulting in a total of 600 restorations. Standardized clinical photography protocols, including consistent camera positioning, fixed shooting distances, controlled lighting conditions, standardized patient positioning, and intraoral scanning protocols, were applied for data acquisition. Linear measurements were obtained from virtual designs and corresponding fabricated restorations. Measurement reliability was assessed using the intraclass correlation coefficient. Statistical analyses were performed to evaluate dimensional deviations and the effects of the software platform and fabrication method (*α* = 0.05).

**Results:**

Statistically significant differences were identified between virtual designs and fabricated restorations; however, the magnitude of these deviations remained small. The manufacturing method demonstrated a greater influence on dimensional discrepancies than the digital smile design software platform. No statistically significant differences were observed between the two software platforms when standardized digital workflow conditions were applied. Measurement reliability analysis revealed high intraclass correlation coefficients.

**Conclusions:**

The fabrication method significantly influenced the 2D accuracy of provisional laminate veneer restorations, with additively manufactured restorations demonstrating smaller deviations in linear measurements compared to milled restorations. No significant difference was observed between the digital smile design software platforms.

## 1 Introduction

The growing integration of digital technologies into restorative and prosthetic dentistry has significantly transformed treatment planning and fabrication workflows, improving procedural standardization and planning predictability. Within this framework, the digital smile design (DSD) concept integrates standardized photography, facial analysis, and digital modeling into a reproducible workflow in which predefined reference lines, facial landmarks, and standardized acquisition parameters are applied to ensure reproducibility and reliability in data acquisition and digital planning that supports diagnosis, interdisciplinary communication, and esthetic treatment planning [1, 2].

DSD has become an integral component of contemporary esthetic dentistry by facilitating visualization of proposed treatment outcomes and enhancing communication among the clinician, dental technician, and patient [3, 4]. In addition, digital planning facilitates the design and evaluation of diagnostic mock‐ups and provisional restorations within contemporary digital workflows, supporting esthetic preview and treatment guidance [5, 6]. Provisional trial restorations allow evaluation of tooth proportions and emergence profiles before definitive treatment, serving as a physical representation of digitally planned designs within the restorative workflow [5, 6, 7, 8].

Among currently available digital platforms, Smile Creator (Exocad GmbH, Darmstadt, Germany) and Smilecloud (Dentcof Clinic, Timișoara, Romania) represent two DSD systems frequently used in esthetic treatment planning. Smile Creator enables a direct three‐dimensional (3D) workflow by integrating intraoral scan data with extraoral facial references, allowing real‐time control of tooth morphology, incisal edge position, and arch form in relation to facial landmarks [9]. In contrast, Smilecloud initiates planning from two‐dimensional (2D) facial photographs and subsequently converts the proposed design into a 3D digital model that can be merged with intraoral scan data, while its predefined tooth libraries and cloud‐based communication tools support interdisciplinary collaboration [10].

Since DSD relies on the accurate transfer of facial and dental records, standardized clinical photography, obtained using fixed camera positioning, controlled lighting conditions, and standardized patient orientation and intraoral scanning, constitutes an essential component of the workflow. High‐resolution photography enables reproducible capture of facial reference lines and smile characteristics, facilitating precise superimposition of dental data onto facial images. Intraoral scanners provide accurate digital impressions and efficient data transfer, allowing seamless integration with computer‐aided design software for virtual planning and fabrication. Together, standardized photography, performed under standardized acquisition conditions, and intraoral scanning contribute to minimizing cumulative registration errors across the digital workflow [10, 11].

Parallel to advances in digital planning software, manufacturing technologies for provisional restorations have also evolved. Additive manufacturing techniques, particularly stereolithography (SLA), fabricate restorations through layer‐by‐layer polymerization of photosensitive resins, enabling high production resolution and reproducible surface characteristics [12, 13, 14]. However, the 2D accuracy of SLA‐fabricated restorations may be influenced by multiple process‐related factors, including resin properties, build orientation, layer thickness, and post‐curing procedures, all of which require careful standardization to support reliable transfer of digital design data into physical restorations [15, 16, 17].

In contrast, computer‐aided design and computer‐aided manufacturing (CAD/CAM) milling represents a subtractive fabrication approach in which restorations are milled from industrially polymerized polymethyl methacrylate blocks. The subtractive production process enables controlled material removal through predefined tool paths and consistent material properties, contributing to the dimensional reproducibility of provisional restorations. Given the fundamental differences between subtractive and additive production principles, manufacturing method selection may influence how digitally planned restorations are physically realized [15, 16, 18]. In this context, previous studies have indicated that manufacturing‐related factors may influence the accuracy of digitally fabricated restorations [19, 20].

Despite the widespread adoption of DSD software and advanced manufacturing technologies, studies that jointly evaluate the influence of digital design platforms and fabrication methods on 2D accuracy within a complete digital workflow remain limited. A previous study compared trial restorations fabricated from virtual planning using SLA printing and CAD/CAM milling and reported superior dimensional accuracy based on linear measurements for SLA‐fabricated restorations across most evaluated parameters [16]. Other investigations have similarly demonstrated that manufacturing method influences the accuracy of provisional restorations [15, 16, 21, 22]. In addition, 3D evaluation methods, such as STL file superimposition and RMS deviation analysis, have also been reported for assessing dimensional discrepancies [23, 24]. However, most existing studies have been conducted within a single digital design environment, limiting the ability to distinguish whether observed dimensional discrepancies arise from the manufacturing process itself or from the digital design stage.

Therefore, the aim of the present study was to evaluate the 2D accuracy of provisional laminate veneer restorations designed using two different DSD software platforms, Smile Creator and Smilecloud, and fabricated using two different manufacturing methods: 3D printing and CAD/CAM milling. The research hypothesis of this study was that there would be differences in the 2D accuracy of provisional laminate veneer restorations depending on the DSD software and the manufacturing method.

## 2 Materials and Methods

### 2.1 Participants

The study was conducted between June 2024 and September 2025 at a university‐based dental clinic and included a total of 25 adult participants aged between 18 and 45 years who presented with esthetic concerns. All participants were required to be in good general health, present with stable periodontal conditions and adequate oral hygiene, and exhibit dissatisfaction with dental esthetics. Following clinical examination, inclusion criteria further required the presence of a complete set of maxillary anterior teeth without extensive restorations or severe intrinsic discoloration, with provisional laminate veneer restorations planned for all six maxillary anterior teeth, and without evident anterior crowding.

Participants presenting with untreated carious lesions, active periodontal disease, missing maxillary anterior teeth, severe malocclusion, or inadequate oral hygiene were excluded from the study.

A priori power analysis was performed using G*Power software (version 3.1.9.6) to determine the required sample size for the study. The calculation was based on dimensional accuracy data obtained from linear measurements reported by Ortensi et al. in the study titled “Accuracy of trial restorations from virtual planning: A comparison of two fabrication techniques” and was conducted using the following parameters: a significance level of *α* = 0.05, a statistical power of 0.95, and an effect size of *f* = 0.338 for repeated‐measures comparisons [16]. Based on these assumptions, the minimum required sample size was calculated as 21 participants. To ensure adequate statistical power and to strengthen the robustness of the analysis, a total of 25 participants were included in the present study.

### 2.2 Clinical Photography

Standardized extraoral and intraoral photographs were obtained for each participant using a mirrorless digital camera system (Sony Alpha 7 III, Sony, Tokyo, Japan) fitted with a 90‐mm macro lens. The camera was positioned on a tripod adjusted to a fixed height to maintain consistent angulation and prevent operator‐dependent variability. For full‐face photographs, images were obtained at a distance of 150 cm, whereas intraoral photographs were captured at approximately 60 cm to ensure appropriate magnification and image sharpness. Participants were seated upright with the Frankfort horizontal plane aligned parallel to the floor to ensure standardized head orientation.

**FIGURE 1:**
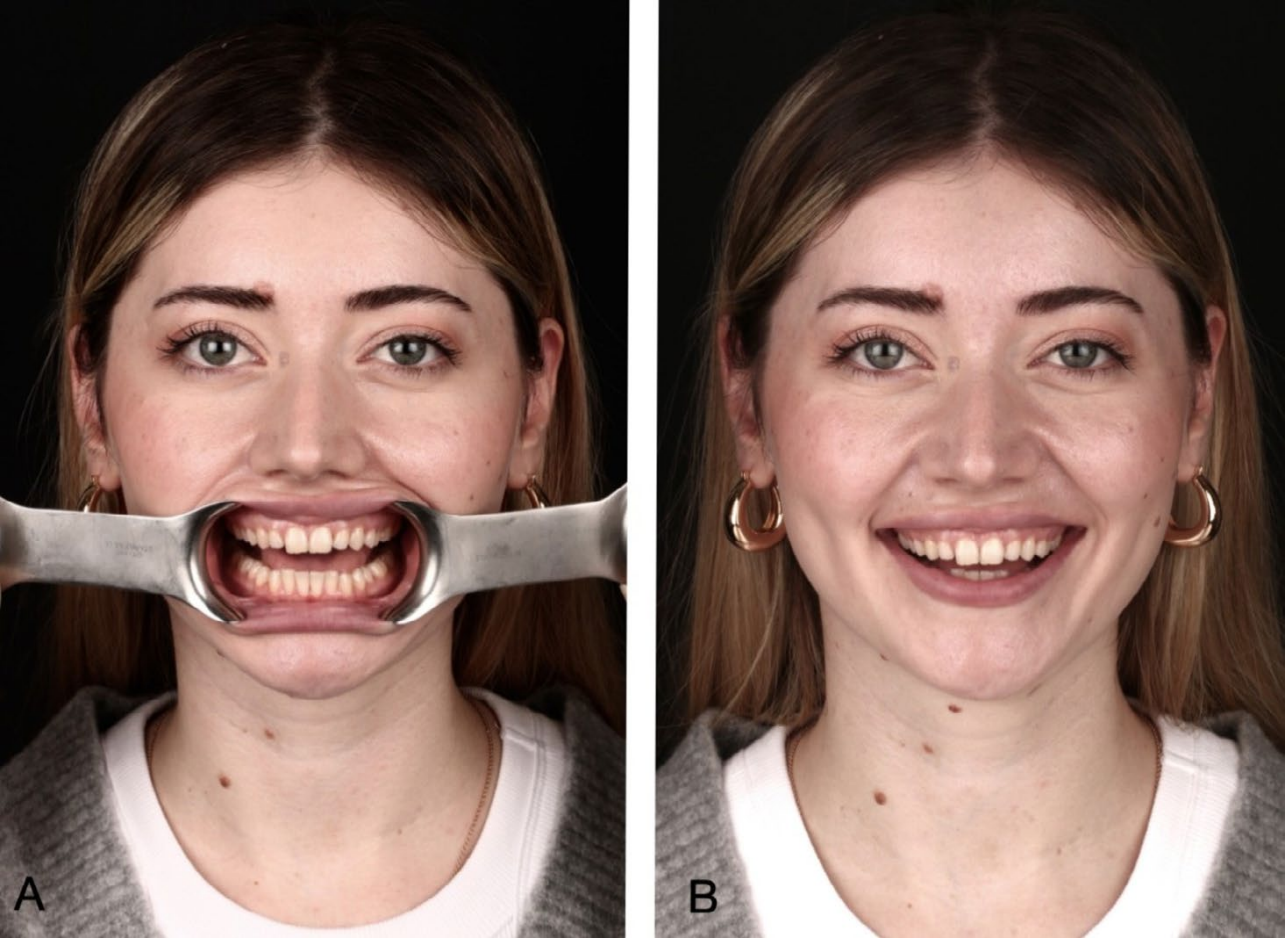
Standardized clinical photography protocol. (A) Retracted portrait photograph. (B) Full‐face portrait photograph with maximum smile.

Illumination was provided by a studio flash unit (Integra, Hensel, Würzburg, Germany) positioned at a 45° angle relative to the facial midline to ensure homogeneous lighting, minimal shadow formation, and accurate color reproduction. Standardized facial photographs were obtained under both retracted and non‐retracted conditions. Retracted images were captured with the lips displaced laterally and the arches slightly separated to allow assessment of the relationship between the pupillary line and the occlusal plane. Non‐retracted photographs were recorded during a natural smile to document esthetic characteristics, including the harmony between the incisal edges and the curvature of the lower lip, smile contour, and buccal corridor display (Figure 1A,B).

### 2.3 Intraoral Scanning

After the photographic documentation, digital impressions of both arches were captured using an intraoral scanner (TRIOS 3, 3Shape A/S, Copenhagen, Denmark). All scans were performed by a single experienced operator to avoid inter‐operator variability. Before scanning, the teeth were gently cleaned and air‐dried to remove excess saliva and plaque, ensuring adequate surface clarity for optical capture. Participants remained seated in an upright position during the procedure, and the scans were obtained under consistent ambient lighting conditions.

The maxillary and mandibular arch scans were obtained according to manufacturer recommendations. All digital impressions were reviewed immediately, and any areas showing distortions or missing information were rescanned to obtain complete and accurate 3D models.

### 2.4 DSD Workflows

**FIGURE 2:**
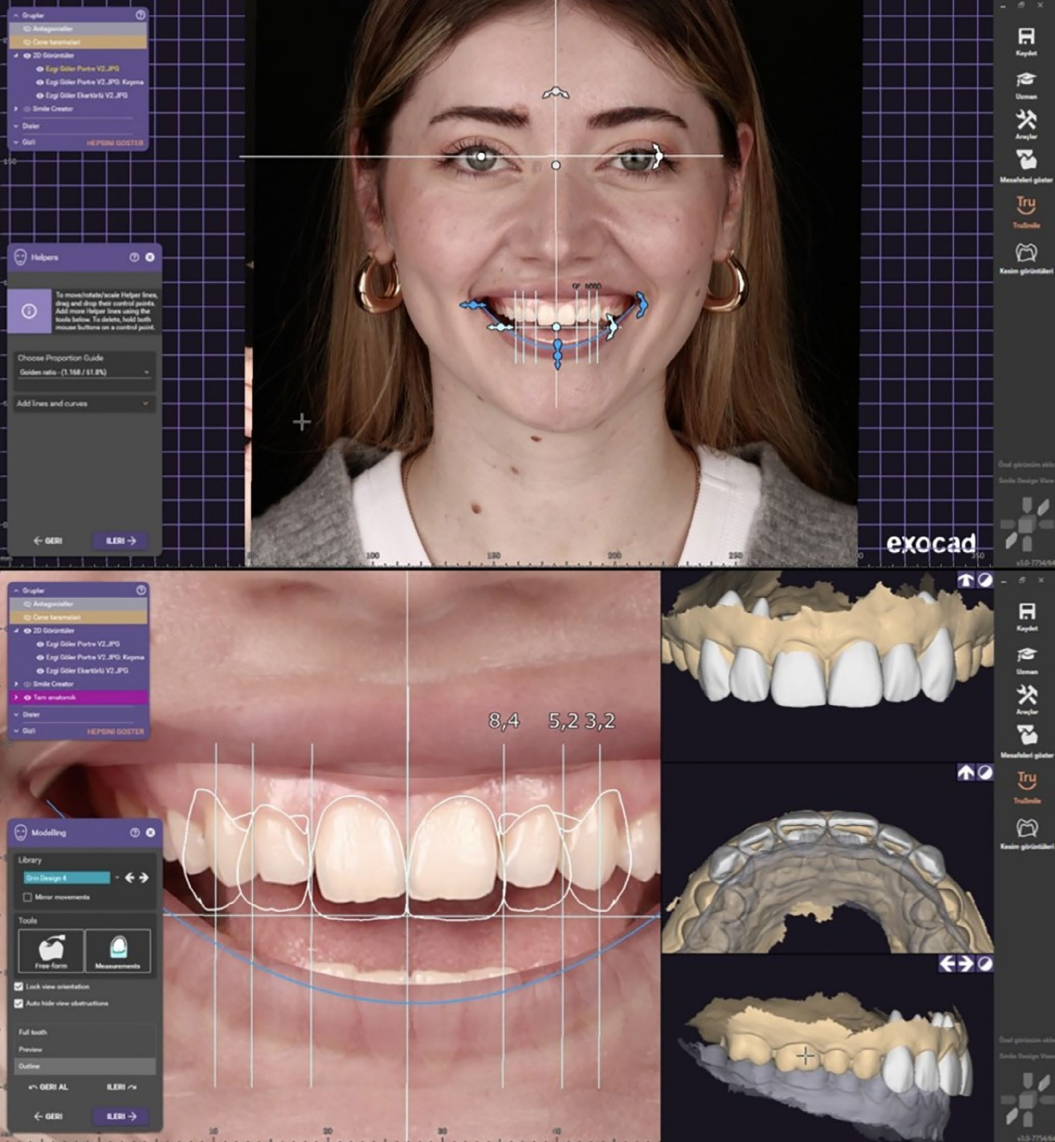
Digital smile design workflow using the Smile Creator software.

The extraoral photographs and intraoral scan files obtained from each participant were imported into two distinct DSD systems: Smile Creator (Exocad GmbH) and Smilecloud (Dentcof Clinic). Smile Creator operates within a fully 3D environment, enabling the integration of facial images with maxillary and mandibular scan data. Using its alignment and calibration tools, facial reference planes are superimposed onto the digital dental arches, based on anatomical landmarks including the interpupillary line, facial midline, and incisal edge position, enabling the alignment of extraoral photographs with intraoral scan data, allowing individualized adjustment of tooth dimensions, proportions, and spatial positioning in accordance with the patient's facial morphology. This direct 3D workflow enables continuous visual oversight of the virtual planning process and facilitates the transfer of the digital design to the fabrication stage (Figure 2).

**FIGURE 3:**
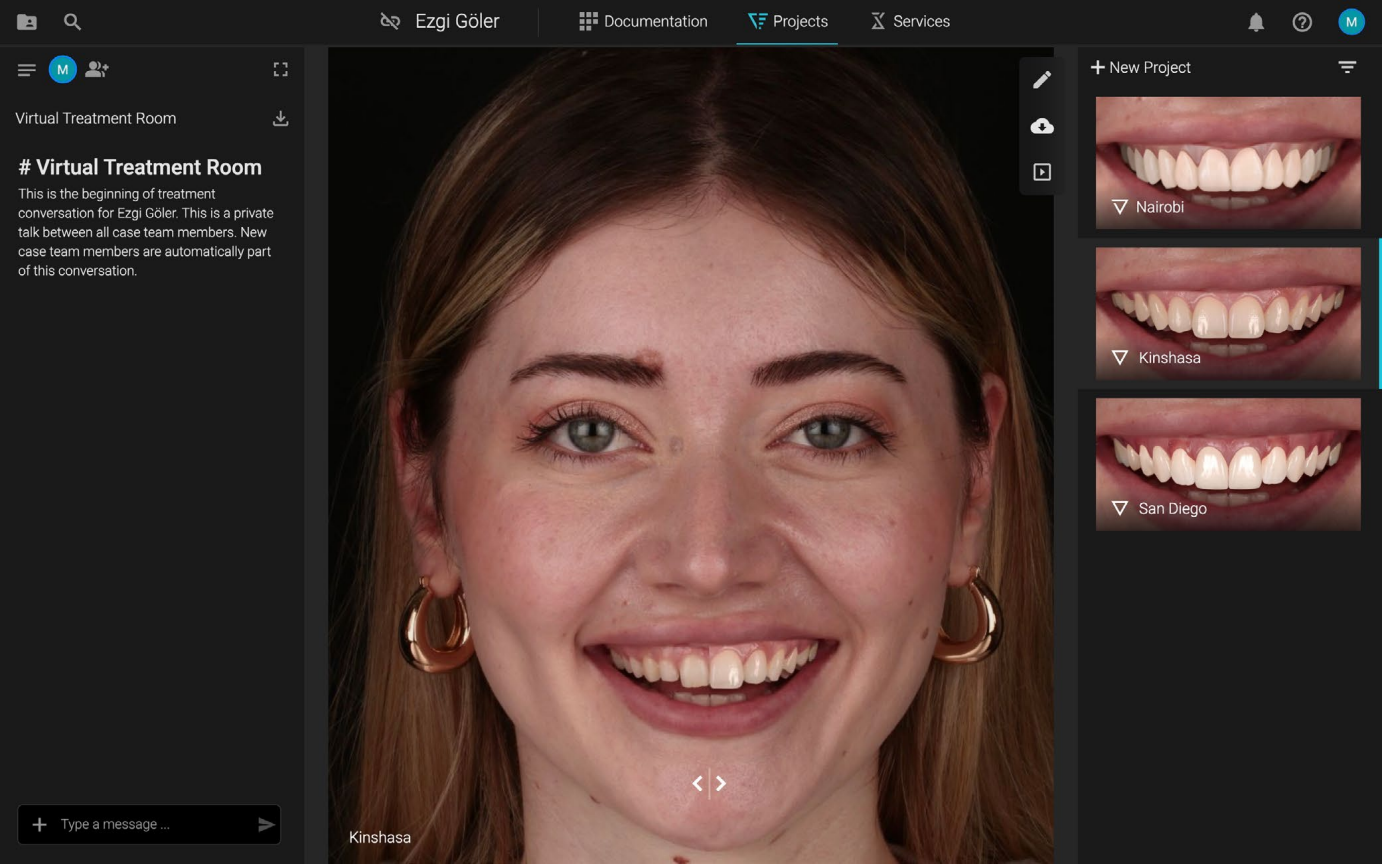
Digital smile design workflow using the Smilecloud software.

Conversely, Smilecloud begins the workflow in a 2D format. At the initial stage, only standardized facial photographs are required, and the software automatically proposes a preliminary smile frame based on anatomical and esthetic reference points. The platform incorporates an extensive library of esthetic tooth shapes, from which templates can be selected and customized (Figure 3). After the 2D arrangement is completed, the system converts the design into a 3D virtual tooth model and exports the data in STL or OBJ format. These files were subsequently imported into a dental CAD software program (Exocad DentalCAD), where the generated 3D designs were aligned with the patient's intraoral scans to complete the virtual restorations.

For each participant, provisional veneer restorations were digitally designed for the six maxillary anterior teeth using both smile design platforms. Two distinct digital design sets were therefore created per participant using the two smile design platforms. Each digital design was subsequently fabricated using the two planned manufacturing methods, with each restoration modeled on an individual tooth basis to ensure accurate morphological control. Following fabrication by the two different manufacturing methods, four restoration sets were obtained per participant, yielding 24 provisional veneer restorations per participant (6 teeth × 2 software platforms × 2 manufacturing methods) and a total of 600 restorations across the 25 participants included in the study.

### 2.5 Fabrication of Restorations

**FIGURE 4:**
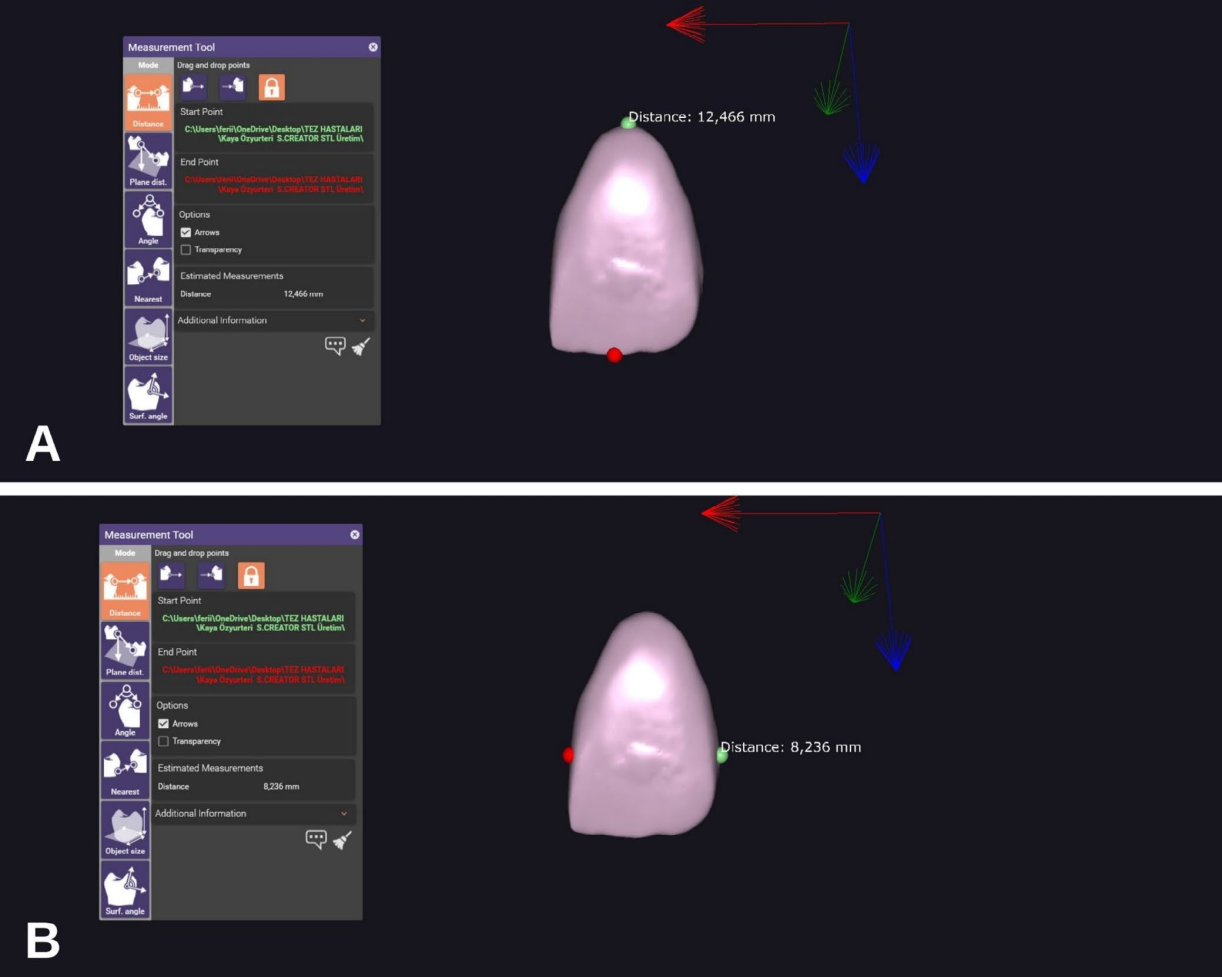
Virtual linear measurement procedure performed on the designed laminate veneer restorations within the CAD software environment. (A) Measurement of inciso‐gingival height. (B) Measurement of mesio‐distal width.

Before the fabrication process, linear measurements were obtained on the virtual veneer designs using predefined reference points (Figure 4A,B). For each veneer restoration, the inciso‐gingival height (distance between the incisal edge and the most cervical point of the restoration) and the mesio‐distal width (distance between the widest points of the crown) were measured on the virtual models. These virtual measurements served as baseline data for subsequent physical comparison. After completion of the digital design procedures and virtual linear measurements, all finalized digital datasets were exported as STL files and fabricated using both additive and subtractive manufacturing workflows.

In the additive fabrication workflow, the restorations were fabricated using an SLA 3D printer (Phrozen Sonic Mini 4K, Phrozen Tech, Hsinchu, Taiwan) with a ceramic‐filled, light‐curable provisional resin material (Alias Dental Temp MHF A1, Dentona AG, Dortmund, Germany). The printing parameters were standardized and kept constant for all specimens. A layer thickness of 50 μm was used. The restorations were printed at an angled orientation, and support structures were placed on the incisal and vestibular surfaces using medium‐sized supports. The exposure time was set at 4.5 s, with a bottom exposure time of 50 s applied for the initial layers.

After printing, the restorations were cleaned in isopropyl alcohol (≥ 96%), air‐dried, and post‐cured in a UV polymerization chamber (CureM U102H, Graphy, Seoul, Korea) at 60°C for 20 min. Subsequently, support structures were removed, and surface finishing and polishing procedures were carried out.

For the subtractive workflow, the same STL datasets were transferred to a five‐axis milling system (inLab MC X5, Dentsply Sirona, Bensheim, Germany) and provisional restorations were milled from PMMA blocks. The milling process was executed using manufacturer‐recommended spindle speed, feed rate, and tool‐path parameters to ensure consistent dimensional replication. Following the milling procedure, the restorations were separated from the milling blocks by removing the connectors. Surface finishing and polishing were performed in accordance with the manufacturer's instructions, and the restorations were subsequently stored until linear measurement and intraoral try‐in.

### 2.6 Measurement Protocol

**FIGURE 5:**
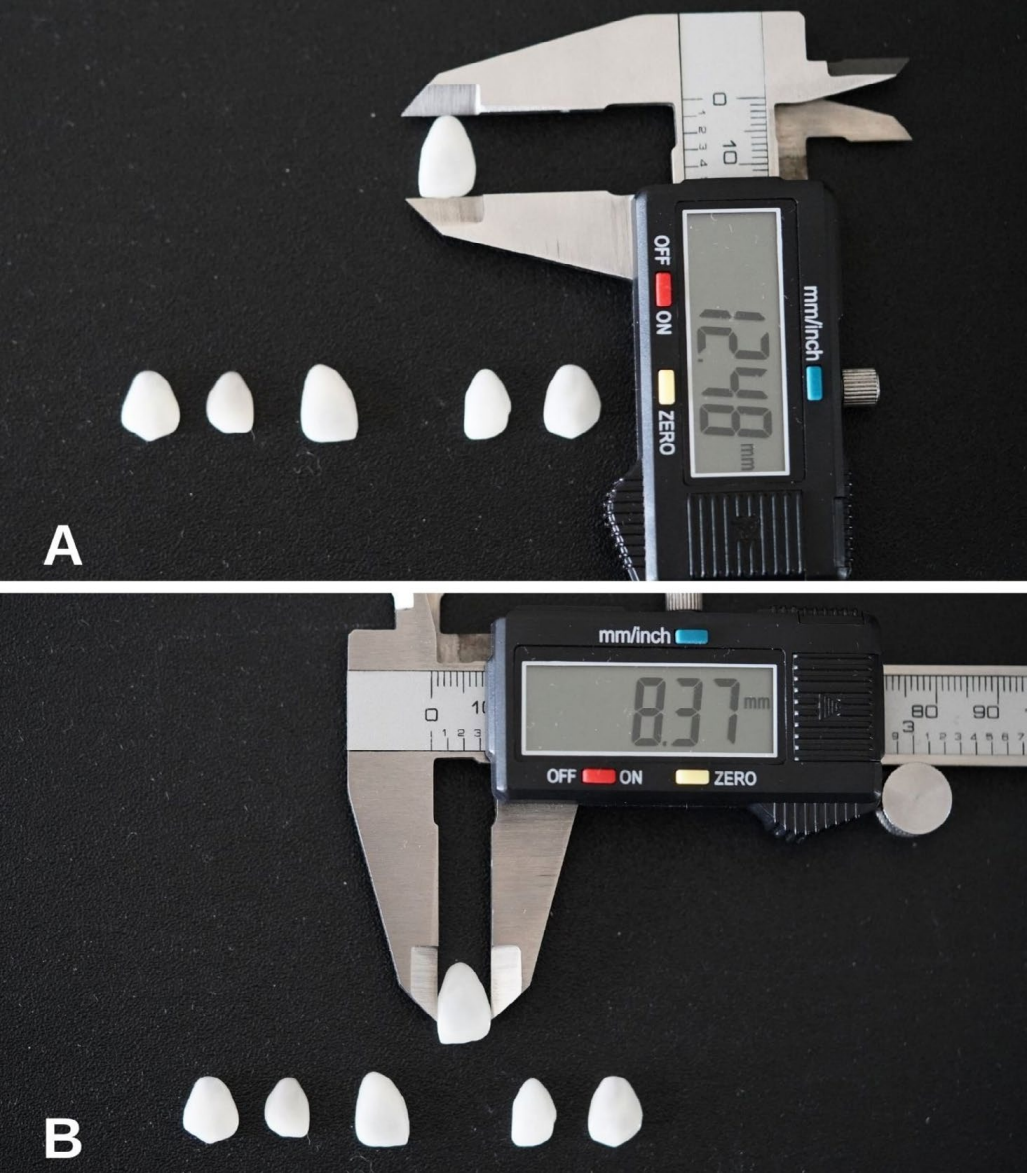
Linear measurement of fabricated provisional laminate veneer restorations using a calibrated digital caliper. (A) Measurement of inciso‐gingival height. (B) Measurement of mesio‐distal width.

After completion of the fabrication procedures, all physical provisional laminate veneer restorations were subjected to linear measurements using a high‐precision digital caliper (Maurer Präzisionsmesszeuge GmbH, Germany) (Figure 5A,B). All measurements were performed by a single calibrated examiner to ensure measurement standardization. The anatomical reference points used for both virtual and physical measurements were predefined and consistently applied across all specimens. The inciso‐gingival height was measured as the distance between the most apical point of the cervical margin (zenith) and the most prominent point of the incisal edge. The mesio‐distal width was defined as the maximum horizontal distance between the most prominent mesial and distal contour points of the restoration. The instrument complies with ISO 13385‐1 standards, ensuring high measurement accuracy, traceability, and repeatability. All linear measurements were recorded according to the measurement resolution of the digital caliper (0.01 mm), and the number of significant figures was determined accordingly to avoid overestimation of precision. The linear measurement values obtained from the fabricated restorations were then directly compared with the corresponding measurements recorded on the virtual designs to evaluate 2D accuracy and deviations occurring across the entire workflow. All measurements were performed three times by the same examiner at different time points, and the mean value of the three measurements was used for statistical analysis. Measurement reliability was evaluated based on 150 repeated measurements obtained from randomly selected restorations within the study sample, all performed by the same examiner.

**FIGURE 6:**
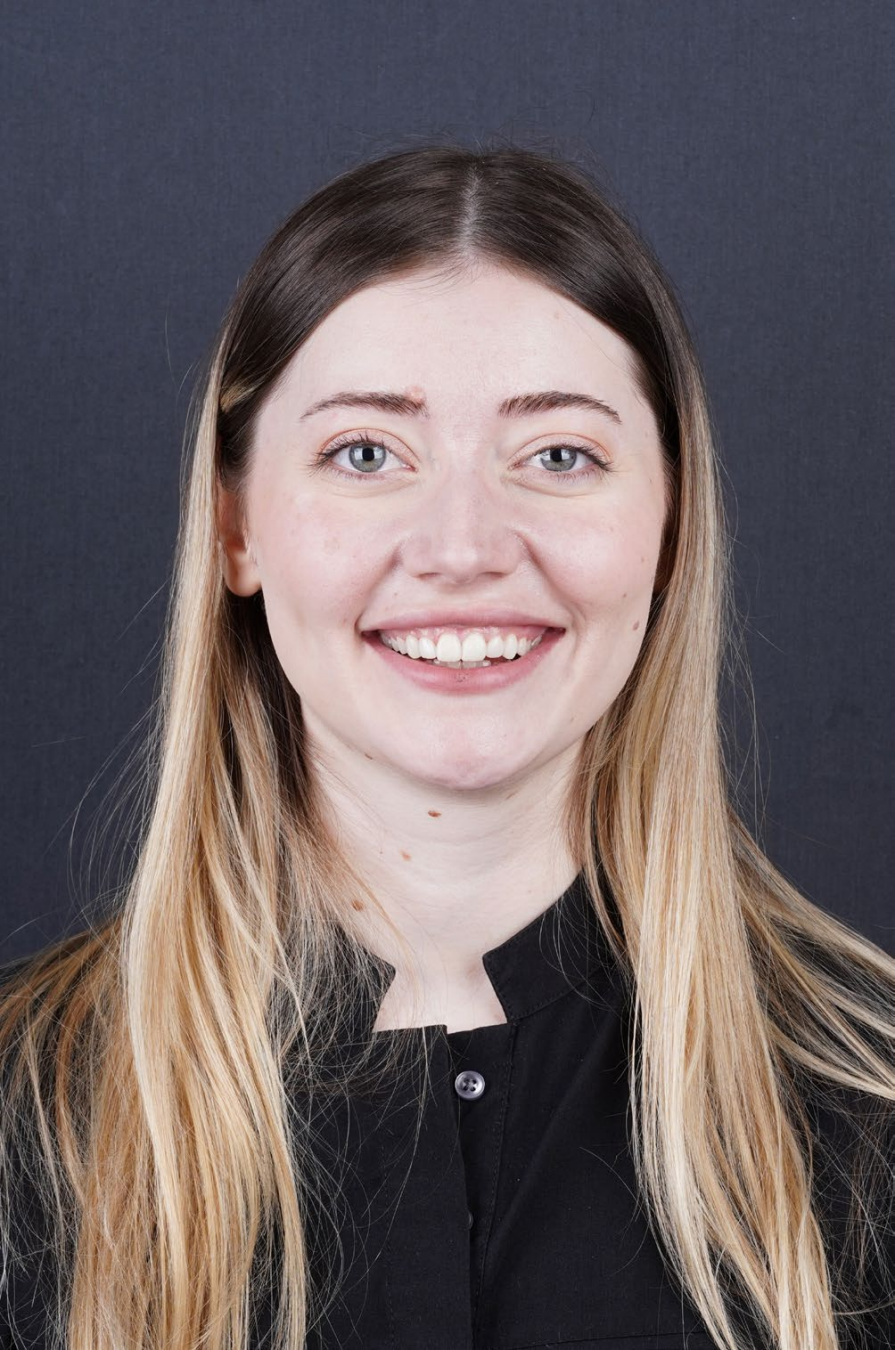
Extraoral frontal view during the clinical try‐in of 3D‐printed provisional laminate veneer restorations.

**FIGURE 7:**
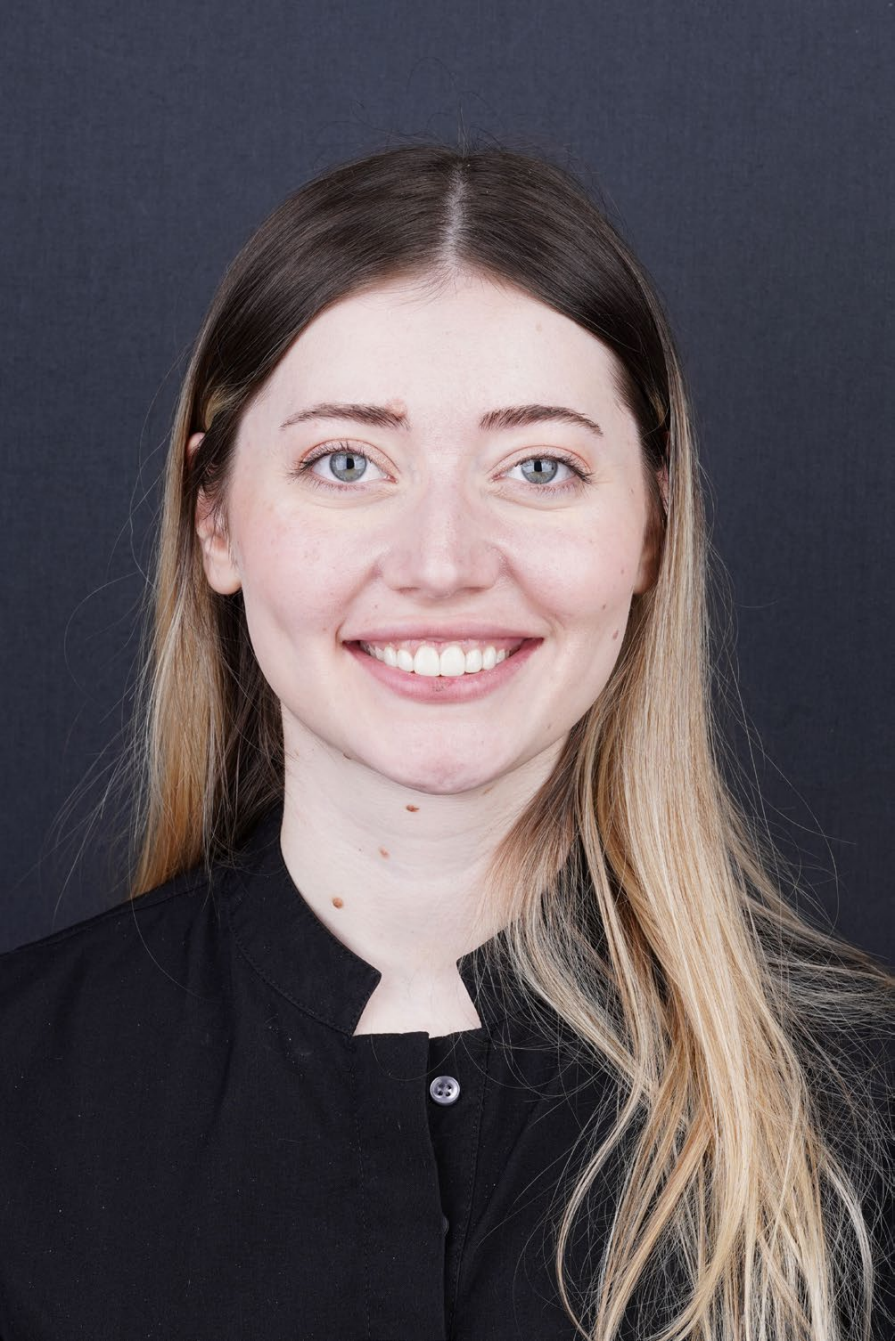
Extraoral frontal view during the clinical try‐in of CAD/CAM‐milled provisional laminate veneer restorations.

Following linear measurements, all provisional laminate veneer restorations were intraorally placed temporarily for visual inspection. Photographic documentation was then performed using standardized extraoral clinical photography under controlled lighting conditions, with consistent camera positioning and standardized patient positioning, following the same protocol described for the initial clinical photographs (Figures 6 and 7). No functional, marginal, occlusal, or esthetic outcome assessments were performed during the intraoral placement procedure.

### 2.7 Statistical Analysis

The statistical analyses were conducted using SPSS software (IBM SPSS Statistics for Windows, Version 28.0; IBM Corp., Armonk, NY, USA). Descriptive statistics were expressed as mean, standard deviation, median, minimum, maximum, frequency, and percentage values. The normality of data distribution was evaluated using the Kolmogorov–Smirnov and Shapiro–Wilk tests. The Friedman test was used to analyze dependent quantitative variables, and pairwise comparisons were performed using the Wilcoxon signed‐rank test. Measurement reliability was assessed using the intraclass correlation coefficient (ICC) for intra‐operator reliability based on repeated measurements (*n* = 150), using a two‐way mixed‐effects model with a consistency definition and a 95% confidence interval. The level of statistical significance was set at *p* < 0.05.

## 3 Results

For both DSD platforms, statistically significant differences were detected between the 3D design and the 3D‐printed restorations for mesio‐distal width and inciso‐gingival height measurements (*p* < 0.05). Despite these differences, intraclass correlation analysis demonstrated a high level of measurement reliability between the 3D design and the corresponding 3D‐printed restorations for both Smile Creator and Smilecloud (ICC > 0.99), where the ICC values represent the agreement between the virtual designs and the 3D‐printed restorations (Table 1).

**TABLE 1:** Comparison of linear measurements between virtual designs and 3D‐printed provisional laminate veneer restorations designed using two digital smile design platforms.

	3D design		3D printing		*p*	ICC	
	Mean ± SD	Median (min–max)	Mean ± SD	Median (min–max)		*r*	*p* ^ICC^
Smile Creator							
Mesio‐distal width (mm)	8.26 ± 1.01	8.40 (5.30–10.70)	8.25 ± 1.03	8.40 (5.30–11.00)	0.002	0.997	< 0.001
Inciso‐gingival height (mm)	9.69 ± 1.08	9.80 (7.00–12.30)	9.71 ± 1.08	9.80 (7.00–12.20)	< 0.001	0.998	< 0.001
Smilecloud							
Mesio‐distal width (mm)	8.24 ± 0.99	8.50 (5.40–10.10)	8.23 ± 1.00	8.50 (5.40–10.10)	0.001	0.998	< 0.001
Inciso‐gingival height (mm)	9.81 ± 1.12	9.90 (6.30–12.30)	9.82 ± 1.13	9.80 (6.50–12.30)	< 0.001	0.998	< 0.001

*Note:* Values are presented as mean ± standard deviation and median (minimum–maximum). The Wilcoxon signed‐rank test was used for pairwise comparisons. ICC represents the agreement between linear measurements obtained from the virtual designs and the corresponding 3D‐printed restorations. Statistical significance was set at *p* < 0.05.

Abbreviation: ICC, intraclass correlation coefficient.

Statistically significant differences were also observed between the 3D design and the CAD/CAM‐milled restorations for both width and height measurements across both DSD platforms (*p* < 0.05). Nevertheless, ICCs indicated high measurement reliability between the 3D design and the CAD/CAM‐milled restorations for both Smile Creator and Smilecloud (ICC > 0.99), where the ICC values represent the agreement between the virtual designs and the CAD/CAM‐milled restorations (Table 2).

**TABLE 2:** Comparison of linear measurements between virtual designs and CAD/CAM‐milled provisional laminate veneer restorations designed using two digital smile design platforms.

	3D design		CAD/CAM milling		*p*	ICC	
	Mean ± SD	Median (min–max)	Mean ± SD	Median (min–max)		*r*	*p* ^ICC^
Smile Creator							
Mesio‐distal width (mm)	8.26 ± 1.01	8.40 (5.30–10.70)	8.50 ± 1.02	8.70 (5.60–10.80)	< 0.001	0.997	< 0.001
Inciso‐gingival height (mm)	9.69 ± 1.08	9.80 (7.00–12.30)	9.92 ± 1.09	10.00 (7.10–12.50)	< 0.001	0.994	< 0.001
Smilecloud							
Mesio‐distal width (mm)	8.24 ± 0.99	8.50 (5.40–10.10)	8.46 ± 0.99	8.70 (5.80–10.30)	0.002	0.997	< 0.001
Inciso‐gingival height (mm)	9.81 ± 1.12	9.90 (6.30–12.30)	10.04 ± 1.15	10.10 (6.70–12.50)	< 0.001	0.997	< 0.001

*Note:* Values are presented as mean ± standard deviation and median (minimum–maximum). The Wilcoxon signed‐rank test was used for pairwise comparisons. ICC represents the agreement between linear measurements obtained from the virtual designs and the corresponding CAD/CAM‐milled restorations. Statistical significance was set at *p* < 0.05.

Abbreviation: ICC, intraclass correlation coefficient.

When dimensional deviations were compared according to the fabrication method, CAD/CAM‐milled restorations exhibited significantly greater deviations than 3D‐printed restorations for both width and height measurements (*p* < 0.05). These findings were consistent across both DSD platforms (Table 3).

**TABLE 3:** Dimensional deviations between virtual designs and fabricated provisional laminate veneer restorations according to fabrication method and digital smile design platform.

	3D design/3D printing		3D design/CAD/CAM milling		*p*
	Mean ± SD	Median	Mean ± SD	Median	
Smile Creator					
Mesio‐distal width (mm)	−0.01 ± 0.11	−0.03	0.24 ± 0.10	0.25	< 0.001
Inciso‐gingival height (mm)	0.01 ± 0.12	0.03	0.23 ± 0.16	0.26	< 0.001
Smilecloud					
Mesio‐distal width (mm)	−0.01 ± 0.08	−0.03	0.22 ± 0.11	0.24	< 0.001
Inciso‐gingival height (mm)	0.01 ± 0.09	0.02	0.23 ± 0.12	0.25	< 0.001

*Note:* Values are presented as mean ± standard deviation. The Wilcoxon signed‐rank test was used for pairwise comparisons. Statistical significance was set at *p* < 0.05.

However, no statistically significant differences were observed between Smile Creator and Smilecloud for 3D‐printed restorations with respect to dimensional deviations in mesio‐distal width and inciso‐gingival height (*p* > 0.05). Similarly, for CAD/CAM‐milled restorations, no statistically significant differences were detected between the two DSD software programs for either mesio‐distal width or inciso‐gingival height deviations (*p* > 0.05) (Table 4).

**TABLE 4:** Comparison of dimensional deviations between two digital smile design platforms according to fabrication method.

	Smile Creator		Smilecloud		*p*
	Mean ± SD	Median	Mean ± SD	Median	
3D design/3D printing					
Mesio‐distal width (mm)	−0.01 ± 0.11	−0.03	−0.01 ± 0.08	−0.03	0.917
Inciso‐gingival height (mm)	0.01 ± 0.12	0.03	0.01 ± 0.09	0.02	0.339
3D design/CAD/CAM milling					
Mesio‐distal width (mm)	0.24 ± 0.10	0.25	0.22 ± 0.11	0.24	0.126
Inciso‐gingival height (mm)	0.23 ± 0.16	0.26	0.23 ± 0.12	0.25	0.431

*Note:* Values are presented as mean ± standard deviation. The Wilcoxon signed‐rank test was used for pairwise comparisons. Statistical significance was set at *p* < 0.05.

## 4 Discussion

The present study evaluated the 2D accuracy of provisional laminate veneer restorations designed using two different DSD software platforms and fabricated through two distinct fabrication methods within a fully digital workflow. Overall, the results indicated that the fabrication method played a more decisive role in dimensional deviations than the DSD software selection. The findings of the present study partially support the proposed research hypothesis. While the fabrication method influenced the 2D accuracy of provisional laminate veneer restorations, no differences were observed between the DSD software platforms. The increasing integration of DSD systems into contemporary prosthodontic workflows has highlighted their role during the diagnostic and provisional phases of esthetic rehabilitation. DSD software is generally regarded as a tool that guides tooth proportions, spatial relationships, and esthetic parameters during virtual planning, potentially influencing the characteristics of provisional restorations [1, 2, 3, 4]. At the same time, fully digital workflows have enabled both additive and subtractive manufacturing techniques for fabricating temporary restorations, with each approach characterized by distinct production principles and sources of variation [15, 16]. Although individual components of digital workflows have been extensively studied, the combined evaluation of DSD software platforms and fabrication methods within a standardized workflow remains limited. Accordingly, this study aimed to evaluate DSD software and fabrication methods in combination and to clarify their relative contributions to dimensional deviations in provisional laminate veneer restorations.

The fabrication method was identified as an important factor influencing the 2D accuracy of the provisional laminate veneer restorations evaluated in the present study. Restorations fabricated using the additive manufacturing method exhibited smaller dimensional deviations compared with those produced by CAD/CAM milling. This finding suggests that manufacturing‐related inaccuracies may have a greater impact than those originating from the digital design phase. Consistent with the present findings, Ortensi et al. reported that 3D‐printed (SLA) trial restorations showed closer agreement with virtual designs than CAD/CAM‐milled restorations [16].

The differences observed between additive and subtractive fabrication methods can be attributed to the manufacturing characteristics of the respective processes. In CAD/CAM milling, the reproduction of fine anatomical details may be limited by factors such as milling bur diameter, tool‐path strategies, and material removal characteristics, particularly in thin or highly contoured areas [15, 16, 17, 18]. In contrast, SLA‐based additive manufacturing enables layer‐by‐layer fabrication, which allows closer replication of complex geometries and smoother surface transitions, thereby supporting more consistent dimensional outcomes during the transfer of the virtual design to the physical restoration, as reported in previous studies comparing additive and subtractive manufacturing techniques [15, 16]. In addition, manufacturing‐related variables such as printing parameters, printer resolution, post‐printing polymerization shrinkage, and curing protocols may have contributed to the relatively smaller deviations observed in additively manufactured restorations, as the controlled polymerization process may result in more predictable and consistent dimensional changes across the restoration [12, 13, 14, 22]. Conversely, in subtractive workflows, several studies have highlighted that limitations related to milling precision, tool diameter, and material removal processes may affect the accuracy of CAD/CAM‐milled restorations [15, 16, 19, 20]. In particular, the observed tendency toward larger dimensions in CAD/CAM‐milled restorations may be associated with milling strategy‐related factors, such as tool diameter compensation and limited bur accessibility in fine anatomical regions, which have been shown to influence the accuracy of milled restorations and limit the reproduction of fine anatomical details [16, 20].

When the two DSD software platforms were compared, no statistically significant differences in dimensional deviations were observed, regardless of the fabrication method used. These results suggest that, under standardized conditions of data acquisition, design protocols, and file export, the influence of the DSD software on 2D accuracy is limited. Although Smile Creator and Smilecloud differ in workflow structure and user interface, both platforms ultimately generate comparable digital design data for transfer to the fabrication stage [9, 10]. As previous studies have largely focused on digital workflows or fabrication methods, the specific contribution of different DSD software platforms to 2D accuracy remains insufficiently explored [9, 10]. In this context, the present findings indicate that software selection alone is unlikely to be a dominant determinant of 2D accuracy within a standardized digital workflow.

Although statistically significant differences were observed between the virtual designs and the fabricated restorations, the magnitude of these deviations was relatively small. Such limited dimensional discrepancies may be considered minimal within the context of provisional laminate veneer restorations. Advances in digital technologies enable the detection of very small dimensional variations; however, these do not necessarily translate into practically relevant discrepancies for restorations intended primarily for diagnostic evaluation, esthetic preview, and patient communication during the clinical treatment planning phase [5, 6, 7]. In addition, it should be noted that the present study was limited to linear measurements, which may not fully represent the 3D accuracy of the restorations. Recent studies have emphasized the use of 3D surface comparison methods, such as STL file superimposition and root mean square (RMS) deviation analysis, to provide a more comprehensive evaluation of dimensional discrepancies [23, 24]. Therefore, the findings of the present study should be interpreted within the context of this methodological limitation. Furthermore, although digital calipers are widely used and provide a practical and reliable approach for linear measurements, advanced digital measurement techniques may offer higher measurement precision and can be considered in future studies for a more detailed assessment. In this context, the linear deviations identified in the present study may have limited clinical relevance in terms of outcomes such as esthetic performance and marginal adaptation, given the small magnitude of the observed differences. Minor discrepancies at the level of tenths of a millimeter are unlikely to alter tooth form or visual appearance and may not be clinically perceptible. Such small variations are also unlikely to affect the overall harmony of the smile or the visual integration of the restorations within the dentition. Therefore, while statistically detectable, these deviations should be interpreted with caution in terms of their direct clinical significance. Accordingly, minor dimensional deviations identified after fabrication should be interpreted in relation to the provisional role of these restorations within the clinical workflow rather than as definitive indicators of restorative performance.

Beyond dimensional considerations, the present findings highlight the importance of workflow standardization in digital prosthodontics. DSD workflows involve multiple sequential steps, including clinical photography, intraoral scanning, virtual planning, and data transfer to the manufacturing stage. Variations at any of these steps may cumulatively influence the final outcome [10, 11]. Accordingly, even when statistically detectable deviations are observed, their clinical relevance should be interpreted in the context of overall workflow consistency and the intended clinical role of the provisional restoration. In addition, potential sources of error may arise at different stages of the digital workflow, including variations in image acquisition conditions, intraoral scanning accuracy, software‐based alignment procedures, and data transfer processes. These factors may contribute to cumulative deviations between the virtual design and the fabricated restoration and may partially explain the differences observed in the present study. Therefore, the results should be interpreted considering the cumulative nature of potential inaccuracies across the entire digital workflow rather than attributing deviations to a single stage.

Despite the strengths of the present study, several limitations should be acknowledged. The assessment of 2D accuracy was limited to linear measurements obtained using a digital caliper, which does not allow a comprehensive 3D evaluation of surface deviations or volumetric discrepancies. In addition, the analysis focused on provisional laminate veneer restorations fabricated using specific DSD platforms and fabrication systems, which may limit the generalizability of the findings to other clinical contexts or restorative designs. Furthermore, only a single 3D printer and a single CAD/CAM milling unit were evaluated in this study. As the performance of digital systems may vary among different manufacturers and technologies, the results should be interpreted within the context of the specific systems used. Nevertheless, the application of a standardized clinical protocol, a controlled digital workflow, and consistent fabrication methods across all groups represents a methodological strength and supports the reliability of the comparisons.

## 5 Conclusion

Within the limitations of this study, it was concluded that:

The fabrication method influenced the 2D accuracy of provisional laminate veneer restorations, with additively manufactured restorations demonstrating significantly smaller deviations in linear measurements compared to milled restorations.

No significant difference was observed in the 2D accuracy of provisional laminate veneer restorations between the DSD software platforms.

## Funding

This work was supported by Marmara Üniversitesi (BAP‐11421).

## Ethics Statement

Ethical approval for this study was obtained from the Ethics Committee of the Faculty of Medicine, Marmara University (Protocol no. 09.2024.537). The study was conducted in accordance with the Declaration of Helsinki, and written informed consent was obtained from all participants before inclusion.

## Conflicts of Interest

The authors declare no conflicts of interest.

## Data Availability

The data that support the findings of this study are available on request from the corresponding author. The data are not publicly available due to privacy or ethical restrictions.
